# Bilateral ovarian fibromas with duplex collecting system and ectopic ureter in an 11-year-old girl: a case report with genetic analysis

**DOI:** 10.3389/fped.2026.1765709

**Published:** 2026-02-03

**Authors:** Lei Zhang, Chunhua Dong, Cuihua Yang, Meng Gui

**Affiliations:** 1 Department of Minimally Invasive Urological Surgery, Children’s Hospital Affiliated to Shandong University, Jinan, China; 2 Department of Minimally Invasive Urological Surgery, Jinan Children's Hospital, Jinan, China; 3 Department of Radiology, Children's Hospital Affiliated to Shandong University, Jinan, China; 4 Department of Radiology, Jinan Children's Hospital, Jinan, China; 5 Department of Ultrasound, Children's Hospital Affiliated to Shandong University, Jinan, China; 6 Department of Ultrasound, Jinan Children's Hospital, Jinan, China

**Keywords:** CAKUT, duplex collecting system, ectopic ureter, *FN1*, ovarian fibroma, pediatric case report, *SUFU*

## Abstract

**Background:**

Bilateral ovarian fibromas in children are exceedingly rare. Their coexistence with congenital anomalies of the kidney and urinary tract (CAKUT) has not been reported.

**Case presentation:**

An 11-year-old girl presented with abdominal pain and lifelong urinary dribbling. Imaging revealed bilateral ovarian masses and a left duplex collecting system with a dilated upper-pole ureter inserting ectopically into the posterior urethra. Serum tumor markers including CA125 were within normal limits. No ascites or peritoneal deposits were identified. Laparoscopic bilateral tumor excision preserved normal ovarian tissue; histopathology confirmed ovarian fibromas with low proliferative index. Three months later, laparoscopic excision of the ectopic distal ureteral segment and ipsilateral ureteroureterostomy completely resolved urinary symptoms. Trio whole-exome sequencing identified heterozygous variants in *SUFU* (p.Asp182Asn) and *FN1* (p.Asn649Ile), both inherited from unaffected parents. At 12-month follow-up, the patient had spontaneous menarche with preserved ovarian function.

**Conclusions:**

This unprecedented dual genitourinary presentation expands the clinical spectrum of pediatric ovarian fibromas. Combined *SUFU* and *FN1* variants suggest possible shared mesodermal developmental susceptibility. Fertility-preserving surgery combined with staged urologic reconstruction achieved full functional recovery.

## 1 Introduction

Ovarian fibromas constitute approximately 4% of ovarian neoplasms in adults but are exceptionally rare in children, with bilateral presentation being even more unusual (1, 2). When bilateral fibromas occur in pediatric patients, they often suggest an underlying tumor predisposition syndrome such as Gorlin syndrome, which is associated with pathogenic variants in *PTCH1* or *SUFU* (3, 4). Congenital anomalies of the kidney and urinary tract (CAKUT), including duplex collecting systems and ectopic ureters, are common in pediatric urology (5). Ectopic ureters draining poorly functioning upper moieties can present with continuous dribbling in girls without typical urinary tract symptoms (6). Single-system ectopic ureters represent a special subset with often delayed diagnosis due to associated renal dysplasia (7). To our knowledge, the coexistence of bilateral ovarian fibromas with CAKUT has not been previously reported.

We present an 11-year-old girl with bilateral ovarian fibromas coinciding with duplex collecting system and ectopic ureter. This case is unique because it represents the first reported dual genitourinary presentation combining a rare pediatric ovarian tumor with congenital urinary tract anomaly, potentially linked through shared mesodermal developmental pathways. We describe the clinical presentation, fertility-preserving surgical management, histopathology, and genomic analysis.

## 2 Case description

### 2.1 Patient information

An 11-year-old Chinese girl presented with 3 months of intermittent cramp-like lower abdominal pain occurring every few days and lasting several minutes. She had no fever, vomiting, or dysuria. Since infancy, she had experienced continuous urinary dribbling between normal voiding episodes, occasionally accompanied by nocturnal enuresis. Multiple courses of acupuncture had been attempted without improvement. She had no history of urinary tract infection, hematuria, or trauma. Growth and neurodevelopment were normal. There was no family history of ovarian tumors, basal cell carcinomas, medulloblastoma, or other features suggestive of Gorlin syndrome. Perinatal history was unremarkable.

### 2.2 Clinical findings

Physical examination revealed a well-appearing afebrile child with mild lower abdominal tenderness and no palpable mass. Abdominal examination showed no distension, shifting dullness, or fluid wave suggestive of ascites. External genitalia were normal with Tanner stage I development, and there were no signs of virilization (hirsutism, clitoromegaly, or acne). A tiny orifice near the posterior urethra suggested an ectopic ureteral opening.

### 2.3 Diagnostic assessment

Laboratory evaluation included serum tumor markers and hormonal assessment. Serum CA125 was 18.2 U/mL (normal <35 U/ml), AFP was 2.1 ng/ml (normal <10 ng/ml), and β-hCG was <0.1 mIU/ml (normal <5 mIU/ml). Sex hormone panel revealed prepubertal levels: estradiol 12 pg/ml, testosterone 8 ng/dl (normal prepubertal <20 ng/dl), FSH 3.2 mIU/ml, and LH 1.8 mIU/ml. These findings excluded hormone-secreting tumors such as fibrothecoma or other sex cord-stromal tumors with endocrine activity.

**Figure 1:**
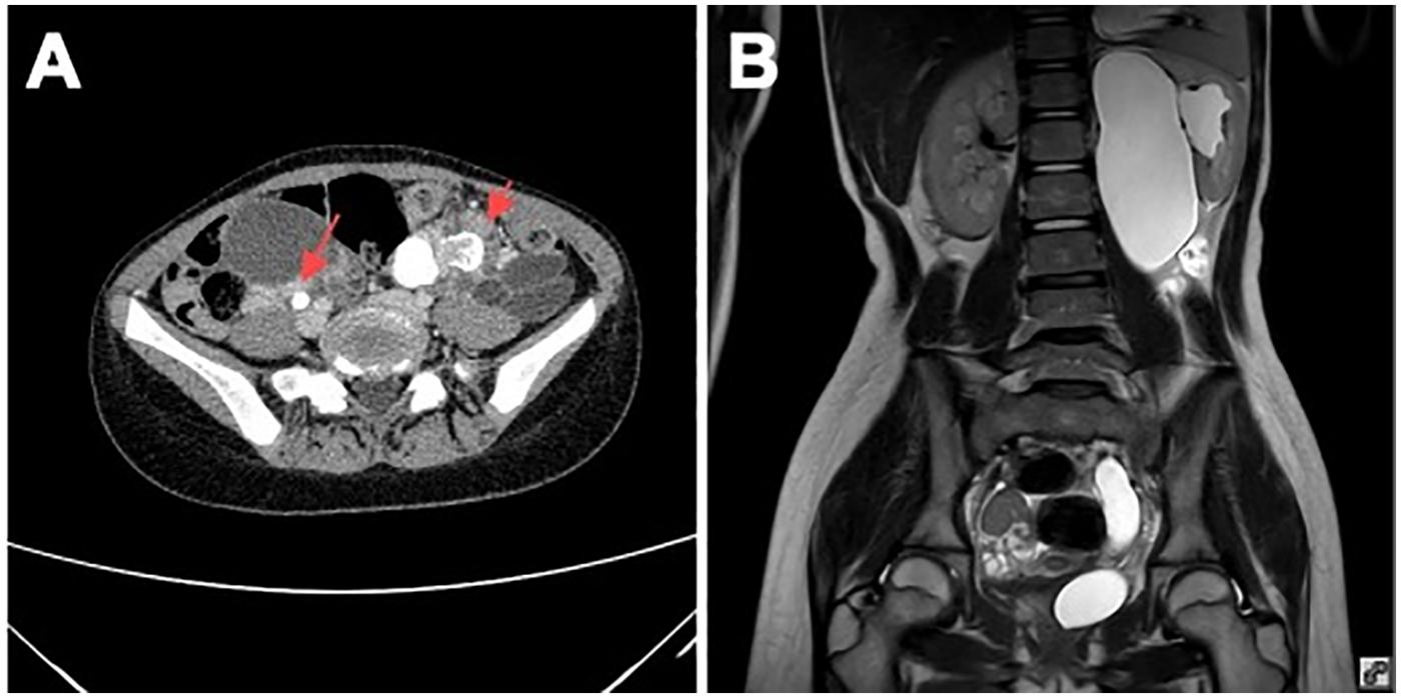
Preoperative imaging findings. **(A)** Contrast-enhanced pelvic computed tomography demonstrating bilateral solid adnexal masses (red arrows) with scattered calcifications. **(B)** Coronal T2-weighted magnetic resonance urography showing bilateral ovarian masses and a duplex left collecting system with hydronephrosis of the upper moiety. The markedly dilated upper-pole ureter (approximately 16 mm diameter) courses caudally toward an ectopic insertion into the posterior urethra.

Pelvic MRI and MR urography revealed bilateral solid ovarian masses and a left duplex collecting system with complete ureteral duplication. The anatomical configuration consisted of two separate renal moieties within a single renal fossa, each with its own ureter: the lower-pole ureter inserted orthotopically into the bladder trigone, while the upper-pole ureter was markedly dilated (approximately 16 mm diameter), coursing caudally and inserting ectopically into the posterior urethra. The upper moiety showed hydronephrosis with cortical thinning; the lower moiety appeared preserved. No peritoneal implants or free fluid were identified (Figures 1A,B). Voiding cystourethrography showed no vesicoureteral reflux. Renal scintigraphy (DMSA) confirmed minimal function (8%) in the left upper moiety, supporting the decision for ureteral reconstruction rather than upper-pole nephrectomy.

### 2.4 Therapeutic intervention

**Figure 2:**
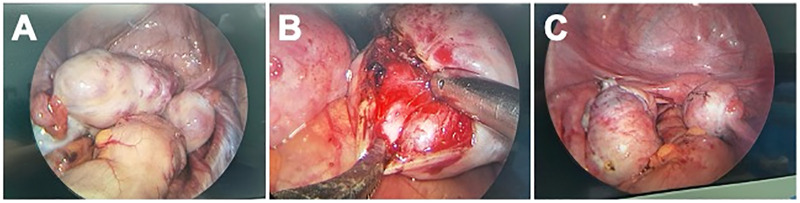
Laparoscopic fertility-preserving surgical management. **(A)** Laparoscopic view showing bilateral enlarged ovaries with firm, nodular surfaces. **(B)** Sharp and bipolar-assisted dissection of the tumor capsule from the residual ovarian cortex. **(C)** Post-excision appearance demonstrating preserved ovarian parenchyma with interrupted absorbable suture reconstruction.

On May 9, 2023, diagnostic cystoscopy followed by laparoscopic bilateral ovarian tumor resection was performed. Cystoscopy demonstrated normal orthotopic ureteral orifices and a pinpoint ectopic orifice on the posterior urethral wall with intermittent urine efflux, confirming the suspected ectopic ureteral opening. Laparoscopy confirmed bilateral enlarged ovaries with firm, nodular surfaces. Inspection of the peritoneal cavity revealed no ascites, peritoneal deposits, or omental involvement. The left duplex ureter was immediately apparent, with the upper branch markedly dilated. Both tumors were completely excised while preserving maximal normal ovarian tissue (Figures 2A–C).

**Figure 3:**
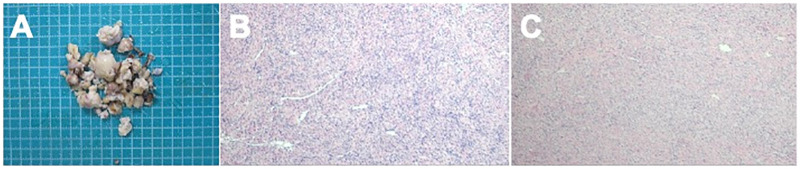
Pathological findings of ovarian fibromas. **(A)** Gross specimen showing multiple nodular tumor fragments with smooth, tan-white, fibrous cut surfaces and focal calcifications. **(B)** Hematoxylin and eosin staining demonstrating densely packed spindle cells arranged in interlacing fascicles within collagen-rich stroma. **(C)** Ki-67 immunohistochemistry showing low proliferative index (3%–5%), consistent with benign sex cord–stromal tumor.

Intraoperative frozen section suggested spindle-cell stromal tumor consistent with ovarian fibroma. Final histopathology confirmed bilateral ovarian fibromas with focal calcification, composed of interlacing bundles of bland spindle cells within collagen-rich stroma. Immunohistochemistry showed diffuse vimentin positivity, focal SMA and inhibin-α positivity, cytoplasmic β-catenin staining, and Ki-67 index 3%–5%, supporting benign sex cord–stromal tumor without thecal or luteinized components (Figures 3A–C).

Three months later (August 30, 2023), the patient underwent staged urologic reconstruction via laparoscopy. The surgical procedure consisted of: (1) mobilization and identification of the dilated ectopic upper-pole ureter; (2) excision of the distal ectopic ureteral segment including its insertion into the posterior urethra; (3) spatulation of the transected upper-pole ureter; (4) end-to-side ureteroureterostomy anastomosing the upper-pole ureter to the ipsilateral normally-draining lower-pole ureter using interrupted 5-0 absorbable sutures; and (5) antegrade placement of a 4.7 Fr double-J ureteral stent across the anastomosis. This approach preserved the functioning lower moiety while redirecting upper-pole drainage through the normal ureteral pathway, eliminating the ectopic insertion responsible for urinary incontinence. The postoperative course was uneventful.

### 2.5 Follow-up and outcomes

Urine dribbling resolved completely following ureteroureterostomy. Follow-up ultrasonography five months later (January 2024) showed stable mild hydronephrosis without progression. At 12-month follow-up (May 2024), the patient had spontaneous menarche, indicating preserved ovarian function. No tumor recurrence was observed. The clinical course is summarized in Table 1.

**Table 1:** Timeline of the patient's clinical course.

Date/Age	Clinical events
Infancy (∼2013)	Onset of continuous urinary dribbling between normal voiding episodes; nocturnal enuresis noted.
2018–2022	Multiple courses of acupuncture attempted without clinical improvement.
February 2023	Development of intermittent cramp-like lower abdominal pain.
May 1, 2023	Initial hospital presentation; pelvic ultrasonography revealed bilateral ovarian masses.
May 3, 2023	Pelvic MRI and MR urography confirmed bilateral ovarian tumors and a left-sided duplex collecting system with an ectopic upper-pole ureter.
May 5, 2023	Preoperative evaluation showed normal tumor markers (CA125, AFP, β-hCG).
May 9, 2023	First surgery: diagnostic cystoscopy and laparoscopic bilateral ovarian tumor excision; intraoperative frozen section suggested spindle-cell stromal tumor.
May 12, 2023	Final histopathology confirmed bilateral ovarian fibromas; Ki-67 proliferation index 3%–5%.
June 2023	Trio whole-exome sequencing performed; heterozygous variants in SUFU and FN1 identified.
August 30, 2023	Second surgery: laparoscopic excision of distal ectopic ureteral segment, ipsilateral ureteroureterostomy, and double-J stent placement.
October 2023	Removal of double-J stent; complete resolution of urinary dribbling.
January 2024	Five-month follow-up: ultrasound demonstrated stable mild hydronephrosis without progression.
May 2024	Twelve-month follow-up: spontaneous menarche achieved; preserved ovarian function confirmed; no evidence of tumor recurrence.

### 2.6 Genetic findings

Trio whole-exome sequencing using peripheral blood samples identified two heterozygous variants of uncertain significance (VUS) in genes involved in embryonic development and extracellular-matrix regulation (Table 2).

**Table 2:** Genetic variants identified by trio whole-exome sequencing.

Gene	Transcript	Variant (c./p.)	Inheritance	gnomAD AF	In silico prediction	Function
*SUFU*	NM_016169.3	c.544G>A (p.Asp182Asn)	Paternal	<0.0001	REVEL 0.82; MutationTaster: disease-causing; ACMG: VUS	Hedgehog signaling; GLI regulation
*FN1*	NM_212482.2	c.1946A>T (p.Asn649Ile)	Maternal	0.00004	PolyPhen-2: 0.958; SIFT: deleterious; ACMG: VUS	ECM assembly; mesodermal differentiation

AF, allele frequency; ACMG, American College of Medical Genetics and Genomics; ECM, extracellular matrix; VUS, variant of uncertain significance.

The *SUFU* missense variant c.544G>A (p.Asp182Asn), inherited from the phenotypically normal father, alters a highly conserved residue within the suppressor domain. This variant is absent from gnomAD (allele frequency <0.0001) and is predicted disease-causing by MutationTaster (REVEL score 0.82). Structural modeling predicted local conformational disturbance that may compromise SUFU's regulatory function in the Sonic Hedgehog pathway (Supplementary Figure S1) (8, 9).

The second variant, c.1946A>T (p.Asn649Ile) in *FN1*, was inherited from the phenotypically normal mother. This variant affects a residue in the fibronectin type III domain, is rare in gnomAD (allele frequency 0.00004), and is predicted pathogenic by PolyPhen-2 (score 0.958) and SIFT (deleterious). Neither parent nor the patient's four-year-old sister showed clinical features. Given that both variants regulate mesodermal differentiation, multilocus genomic variation may underlie this combined ovarian–renal phenotype (10).

## 3 Discussion

This case documents an unprecedented association of bilateral ovarian fibromas with duplex collecting system and ectopic ureter in a child. The clinical presentation, comprehensive imaging, histopathology, and genetic analysis provide insights into potential shared developmental mechanisms.

Bilateral ovarian fibromas in children are rare and often prompt evaluation for tumor predisposition syndromes such as Gorlin syndrome (3, 4). Our patient's heterozygous *SUFU* variant (p.Asp182Asn), though classified as VUS, affects a highly conserved residue. Pathogenic *SUFU* variants cause Gorlin syndrome, characterized by medulloblastoma, basal cell carcinomas, and bilateral ovarian fibromas (8, 9). However, our patient lacks other Gorlin features, and her father (variant carrier) remains asymptomatic, suggesting incomplete penetrance.

The *FN1* variant adds complexity. Fibronectin is essential for mesodermal tissue organization and morphogenesis (11, 12). During embryonic development, both the urogenital ridge (giving rise to kidneys, ureters, and gonads) and gonadal stromal tissues derive from intermediate mesoderm, sharing common developmental origins (13, 14). Combined *SUFU* (affecting stromal proliferation) and *FN1* (affecting extracellular matrix) variants may act synergistically, contributing to this dual phenotype (10).

The staged surgical approach—fertility-preserving ovarian tumor excision followed by ureteroureterostomy—achieved excellent functional outcomes. Management of pediatric ovarian masses emphasizes preservation of normal tissue whenever feasible, as most lesions are benign (15). Risk stratification for malignancy guides surgical planning (16), and minimally invasive approaches including single-incision techniques have demonstrated excellent outcomes (17). Our patient's spontaneous menarche at 12 months demonstrates successful ovarian preservation.

### 3.1 Strengths and limitations

Strengths of this case include comprehensive multimodal evaluation integrating advanced imaging, detailed histopathology, and trio whole-exome sequencing, along with excellent clinical outcomes with documented functional recovery. Limitations include the VUS classification of both variants without functional validation, the single-case design precluding causal inference, and limited long-term follow-up for tumor recurrence risk assessment.

## 4 Patient perspective

The patient's mother reported: “Our daughter had been troubled by urinary leakage since birth. We tried many treatments including acupuncture without success. When she developed abdominal pain, we were worried. The doctors explained that she had both ovarian tumors and a urinary problem, which was very rare. After two surgeries, her leakage completely stopped and she has been doing well. She recently had her first period, which reassured us that the ovarian surgery was successful. We are grateful for the careful surgical approach that preserved her fertility”.

## 5 Conclusion

We report the first case of bilateral ovarian fibromas coexisting with duplex collecting system and ectopic ureter in a child. Identified *SUFU* and *FN1* variants suggest a possible mesodermal developmental link. Laparoscopic fertility-preserving tumor excision combined with staged reconstructive urologic surgery achieved full functional recovery. This case highlights the need for integrated surgical–genetic assessment and lifelong follow-up in children presenting with concurrent reproductive and urinary anomalies.

## Data Availability

The original contributions presented in the study are included in the article/Supplementary Material, further inquiries can be directed to the corresponding author.
