# Supplementary material for: Bilateral ovarian fibromas with duplex collecting system and ectopic ureter in an 11-year-old girl: a case report with genetic analysis
Source: Front Pediatr. 2026 Feb 3;14:1765709. doi: 10.3389/fped.2026.1765709 (PMC12909570; doi:10.3389/fped.2026.1765709)

**Supplementary Material**

Bilateral Ovarian Fibromas with Duplicated Kidney and Ectopic Ureter in an 11-Year-Old Girl: A Case Report with Genetic Analysis

**Supplementary Figure 1 |** *In silico* **structural modeling of the SUFU protein variant.**

**(A)** Overall three-dimensional structure of the wild-type SUFU protein with the suppressor domain highlighted (cyan and magenta regions).

**(B)** Close-up view of Asp182 in the wild-type structure showing stable hydrogen bonds (yellow dashed lines) with neighboring residues including His89, Arg123, Thr121, Gln184, Met185, and Phe197.

**(C)** Overall structure of the SUFU p.Asp182Asn mutant model showing similar global architecture.

**(D)** Local view illustrating disruption and rearrangement of hydrogen bonds following the Asp182Asn substitution. The mutation results in loss of interactions with Arg123 and Gln184, and formation of new bonds with Glu181 and Pro183. These alterations indicate local conformational destabilization that may impair SUFU's regulatory function in the Sonic Hedgehog signaling pathway.

**Methods:** Structural modeling was performed using PyMOL (Schrödinger, LLC) based on the crystal structure of human SUFU (PDB: 4BLD). The p.Asp182Asn variant was introduced using the mutagenesis wizard, followed by energy minimization. Hydrogen bond networks were analyzed and visualized using default parameters.


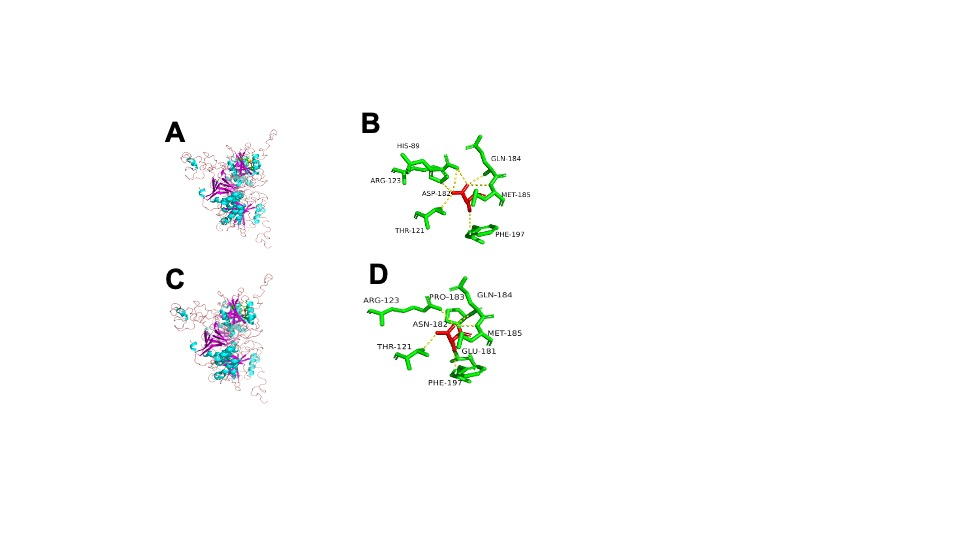

Supplement: Supplementary file 1 [file Supplementaryfile1.docx]
